# “Helping Mentally Ill, a Reward Both in this Life and After”: A Qualitative Study Among Community Health Professionals in Somaliland

**DOI:** 10.1007/s10597-022-01085-8

**Published:** 2023-01-05

**Authors:** Sungkutu Nyassi, Yakoub Aden Abdi, John Minto, Fatumo Osman

**Affiliations:** 1 School of Health and Welfare, Dalarna University, 79188 Falun, Sweden; 2 College of Health Science, Amoud University, Amoud Valley, Borama, Somaliland Somalia; 3 University of the West of Scotland, Paisley, Scotland

**Keywords:** Community mental health, Mental health services, Post-conflict setting, Fragile state

## Abstract

This study aimed to describe the experiences of community mental health workers, predominantly female, nurses and doctors providing community-based mental health services in Borama, Somaliland. A qualitative explorative study using focus group discussions was conducted. Data were collected from three focus group discussions with 22 female community health workers, two medical doctors, and two registered nurses and analyzed using content analysis with an inductive approach. Three main categories were identified from the analysis: (1) bridging the mental health gap in the community; (2) working in a constrained situation; and (3) being altruistic. Overall, the community mental health workers felt that their role was to bridge the mental health gap in the community. They described their work as a rewarding and motivated them to continue despite challenges and improving community healthcare workers’ work conditions and providing resources in mental health services will contribute to strengthening mental health services in Somaliland.

## Background

Mental health problems are highly prevalent worldwide, affecting people in every community and age group across all global regions (Chaulagain et al., 2020). It is estimated that 792 million people live with mental health problems and that one in every 10 people globally experiences a mental health problem (Dattani et al., 2021). Despite substantial research in the prevention, treatment, and promotion of mental health, the translation into real-world effects has largely failed. The global burden of mental health problems has increased dramatically in all countries within major demographic, environmental, and sociopolitical transitions. Human rights violations and abuse among people suffering from mental health problems persist in many countries, with large numbers of people locked away in psychiatric hospitals, prisons, or living on the streets, often without legal protection (Patel et al., 2018).

In low-resource settings, such as parts of Africa, mental health continues to be a significant public health concern, aggravated by a lack of knowledge, institutional neglect, and widespread stigma (Iheanacho et al., 2016; Labinjo et al., 2020). Mental illness is often associated with supernatural causes, such as evil spirits, witchcraft, and is also often seen as a punishment from God for bad behavior (Labinjo et al., 2020). It is also believed that a person with a mental illness is prone to violence, unpredictable, and motivated by evil spirits, many of which are believed to be incurable (Iheanacho et al., 2016; Labinjo et al., 2020).

Over the past three decades, Somalia has suffered from prolonged conflict, displacement, poverty, and famine, all of which have had a long-term impact on the population’s mental health (Ibrahim et al., 2022). Almost all Somalis have experienced or witnessed violence to some extent. Conflict-induced humanitarian crises have affected the social determinants of mental health and the well-being of families and communities, as well as their access to basic services and education (Lindvall et al., 2020). The health infrastructure was destroyed during the war, and many healthcare workers have been displaced and eventually left the country. The mental health service in Somalia is neglected, while the demand is enormous. To close the mental health gap, community-based mental health services engaging non mental health specialists have been proposed by World Health Organization (WHO) due to the lack of human resources, such as psychiatric specialists, particularly in low-income countries (Chaulagain et al., 2020). However, working as a community mental health worker can be one of the most complex and challenging roles in mental health services (Rossi et al., 2012).

The WHO Mental Health Gap Action Program (WHO, 2015) and the Lancet Series on Global Mental Health (Frankish et al., 2018; Patel et al., 2008) highlighted the importance of closing the mental health gap by developing interventions that can be implemented by non mental health specialist. The mental health gap is an action program developed by the WHO, and aims to scale up mental health services for low- and middle-income countries (Campion et al., 2022; Frankish et al., 2018; Patel et al., 2008). Community mental health workers have been identified as a workforce that can tackle the mental health gap as they possess the principles and practices needed to promote mental health for a local population (Thornicroft et al., 2016). Community mental health workers assist in addressing the needs of a population in ways that are accessible and acceptable to the local people, build on the goals and strengths of people who experience mental illnesses, promote a wide network of supports, services, and resources of adequate capacity, and emphasize services that are evidence-based and recovery‐oriented. It has been proposed that community mental health workers should focus on how people can recover from mental illness, thus strengthening people and the support around them (Thornicroft et al., 2016).

However, working as a community mental health worker can be one of the most complex and challenging professions in mental health services delivery (Agyapong et al., 2015; Akol et al., 2018; Rössler, 2012). Studies have reported that health professionals working on community mental health teams suffer from increasing levels of stress and burnout following growing workloads and administrative tasks, as well as a lack of resources. The effects of time management, safety issues, role ambiguity, role conflict, lack of training, lack of proper supervision, poor general working conditions, lack of funding, and resources also have a significant impact on workers’ well-being (Agyapong et al., 2015; Akol et al., 2018; Rössler, 2012).

Somaliland is one of the countries with the highest prevalence of mental health disorders globally, with two out of every five people estimated to be living with a severe mental illness (Rivelli, 2010). People with mental disorders are highly marginalized in the Somali community and people with mental health problems are often isolated at home, held in chains, abused, and sometimes even kept in Ilajs (traditional healing centers) or prisons for years without release (Abdi Abdillahi et al., 2020; Handuleh et al., 2014). Studies have further shown that very few have access to treatment due to stigma and the shortage of trained mental healthcare providers (Handuleh et al., 2014; Leather et al., 2006).

To address the lack of access to mental healthcare in Somaliland, in 2013, members of the Somali diaspora in collaboration with Amoud University College of Health Science in Borama, Somaliland, initiated and mobilized resources to establish a community-based mental health program. The program continued until 2021, when ownership of the project was handed over to Somaliland’s Ministry of Health (Abdi et al., 2021). In the initial phases of the mental health project, maternal, neonatal, and children’s health were included in the program due to the stigma associated with mental illness. This approach increased community acceptance of the project. From 2015, after the community became accustomed to the project and realized its benefits. The findings from the project showed that 202 out of 237 individuals with mental illness were unchained due to the community-based mental health programs implemented in the Awdal region of Somaliland (Abdi et al., 2021).

To date, there is no research on how community mental healthcare providers can support closing the mental health gap in Somalia. Thus, the present study aimed to describe the experiences of community mental health workers in providing community-based mental health services in Borama, Somaliland. Understanding their experiences will be useful in guiding the newly revised mental health policy in Somaliland on how to utilize community mental health providers’ knowledge and resources.

## Methods

### Design and Setting

The study, which was conducted in 2019, used a descriptive qualitative study design using focus group discussions (FGDs) and was part of a project implementing community mental health services in the Awdal region of the self-declared Somaliland. The republic of Somaliland declared independence from Somalia after the civil war’s 1991 and has been stable for two decades during which health infrastructure has been significantly rebuilt. The data were collected in the city of Borama, which is the third-largest town in Somaliland, with a population of 278,000 (United Nations Population Fund, 2014). Borama has one main regional hospital, a teaching hospital and ten Maternal and Child Health facilities. The Borama regional hospital has one psychiatric ward to which all patients with mental health problems are referred. In addition, there are many traditional healers in the community, and many people take their relatives with mental health problems to them.

In 2013, a community-based maternal, neonatal, child, and mental health project was established in a small sector of Borama and slowly expanded to the whole of the town and to two other smaller towns, Baki and Dila (Abdi et al., 2021). The overall project aimed to improve access to essential services (maternal, neonatal, child health, and mental health services) for the population in defined areas in the Awdal region. The current study focused on community health workers engaged with the mental health project. The project started with 10 pretrained FCHWs, one nurse, and one doctor in a small sector of Borama with a population of approximately 10,000 inhabitants. In the context of Somaliland, the FCHWs are lay women who are not required to have any health education, but they are given training in mental health. In this study, the community health workers also included doctors and nurses, in addition to the FCHWs.

In 2015, the project was expanded, recruiting a further 30 FCHWs (total 40), four nurses (total five), one doctor (total two), and one project manager. The services were scaled up to the whole of Borama, Baki, and Dila. Before their deployment, FCHWs received 3 months training in basic knowledge of mental health, and how they could detect people with mental illness and refer them to the hospital. Furthermore, they received skills such as communication, sensitization with the family, raising awareness to reduce the stigma and techniques in counselling. Throughout the project the FCHWs received a monthly recap training from the doctors and nurses in the project. The training of the FCHWs was provided by nurses, doctors in the project as well as through lectures from College of Health Sciences at Amoud University. The FCHWs worked 6 h per day for 6 days per week and were required to visit six families each day. Their activities included identifying people with severe mental disorders, educating the whole family, counseling patients, and distributing medicines to patients in their home environment. Due to financial constraints, from 2018, the team was reduced to 20 FCHWs, three nurses, two doctors, and one project manager until its end in 2021. However, the FCHWs, doctors, and nurses worked as a team (Abdi et al., 2021).

### Participants and Recruitment Procedure

All staff in the community mental health project were eligible to participate and were asked for their oral consent to participate in the study. In total, 26 participants agreed to be enrolled in the study. The 26 participants comprised 22 FCHWs, two medical doctors, and two registered nurses (see Table 1 for participant characteristics).

**Table 1:** Demographic characteristics of the participants

FGD	Variable	Participants (*n* = 26)
1	Gender	25 females (22 FCHW and 2 nurses), 1 male medical doctor
	Age	Mean age 26.5 years. FCHWs ages ranged from 17 to 30 years. Doctors and nurses from 29 to 36 years
	Education	11 participants had university-level education (2 doctors, 2 nurses and 7 FCHW), 14 FCHWs secondary-level, and 1 FCHW intermediate-level.
	Experience in mental health services	2–6 years

### Data Collection

The data were collected using FGDs. A predefined semi-structured interview guide was used (Table 2). The interview guide was not pilot tested because there were no existing community health services conducting similar work to that of the participants we were interviewing. However, the interview guide was discussed with the advisory committee (project leaders, project manager, and coordinators) and the researchers before conducting the FGDs.

**Table 2:** Interview guide

Topic	Questions
Community health worker’s role	Can you tell me about your role as community health workers? What is your role? Can you describe of how a typical day of your work looks like? (Follow-up with what, how, why, why not, whom, when)
Challenges and possibilities	What is that you encounter when working as a community health worker? What are the possibilities working as a community health worker? What are the challenges you encounter from the community? How can you solve these challenges? Can you tell me about the success or non-success in your work? (Follow-up with what, how, why, why not, whom, when)
Community’s view	Traditional versus hospital care, how does the community see that? Any other information?

In total, three FGDs were conducted, with one group consisting of doctors and nurses (FGD3) and two groups with FCHWs (FGD1 and FGD2). Each FGD lasted from 45 to 60 min. All FGDs were audio-recorded and transcribed verbatim in Somali, and the Somali transcriptions were then translated into English by a research assistant who was bilingual with formal qualifications related to language translations and was not part of the project. The translated transcript was checked by the coauthor who conducted the FGDs to ensure the reliability of the translation.

### Data Analysis

All translated and transcribed data were analyzed inductively using manifest content analysis (Elo et al., 2014). Manifest content analysis represents a systematic and objective means of describing and quantifying phenomena in which data can be reduced to concepts that describe the research phenomenon. The inductive approach of content analysis is most suitable when little is known about the content under study as it enables researchers to analyze data in areas in which only limited knowledge exists (Elo et al., 2014). Inductive reasoning was applied to see which themes emerged from the raw data through repeated examination and comparison of the data (Elo et al., 2014). All the transcripts were read thoroughly by the first and last authors to understand the content. Words, phrases, or other text that responded to the aim were highlighted. Open coding was then performed, meaning that the phrases, words, or text were condensed into meaningful units. Next, all the transcripts were coded and grouped into clusters based on their similarities. Each cluster code was used to build a subcategory, which were formed into categories (see Table 3 for an example of the analysis process). All the authors discussed the process of analysis to ensure the credibility and confirmability of the identified categories.

**Table 3:** Example of meaning unit, condensed meaning and condensed units, code, subcategories, and categories

Meaning unit	Condensed unit	Codes	Subcategory	Categories
… We give them awareness about the disease so that they tell us about the sick person, their family, or even their neighbors	Giving information and raising awareness in the family and neighborhood	Awareness in the community	Connecting families to mental health services	Bridging the mental health gap in the community
Several times, I visited to a mother who was hiding her daughter, who was tied with a chain. When the mother came to know that we work for the people with mental illnesses and that many of them take medicines and recover, she contacted us later and received our support. Her daughter’s illness was diagnosed by the doctor as bipolar disorder. She trusted that we only wanted to help her and the daughter.	Families hide and chain their mentally ill members, particularly girls. Making them realize that the existing support can help both the patient and family.	Trust makes the family accept help	Building trust in the community	
The family do not believe that the patient can recover from the mental illness. Family members say to us ‘ leave him, he will never recover from disease’. This is a challenge for us to persuade the family members	Family members do not believe that their relative will recover from the mental illness and it is difficult to persuade the family	Lack of knowledge and stigma might prevent the patient from receiving help	Stigma as a hindrance to accept mental healthcare	Working in a constrained situation
I know that family, they told me that he was bewitched, and he does not need a hospital. They preferred to take the patient to the traditional healers.	The family refuse to take to take relatives to hospital and prefer traditional healers	Believing traditional healers can cure the patient with mental illness	Traditional beliefs as a hindrance to mental healthcare	
There are no supports for the nurse and female community health workers to increase their knowledge. There is no official training except on job training, and this can decrease the motivation of the female health workers. Our salary is very low compared to the work we do. But we want to help these patients. We know they don’t have anyone else.	No training for the female community health workers and nurses. This could impact negatively on their motivation. Low salary in relation to the burden of their work. Motivation through the help the give to the patients.	The job is related to many difficulties and few incentives	The burden of working with mental health problems	Being altruistic
My job is good, the best blessings in both this life and after is to help those who are mentally ill. It is humanly to work for the poor people who have no one helping them	Helping people with mental illness is rewarding particularly when they don’t have anyone who is helping them.	Helping people with mental illness is rewarding	Rewarding job	

### Ethical Consideration

The project received ethical approval from Research Ethical Committee at Amoud University and permission from the Awdal Region Health Officer. The participants were given in Somali both oral and written information about the study and informed that they could withdraw from the study at any time without compromising their jobs. As the nature of this study was not to collect any sensitive or personal information from the participants, only verbal consent was sought. This was also stated in the ethical approval of the project. The data was kept confidentially and only the last author had access to the audios. The interviews were anonymized after transcription.

## Results

**Fig. 1:**
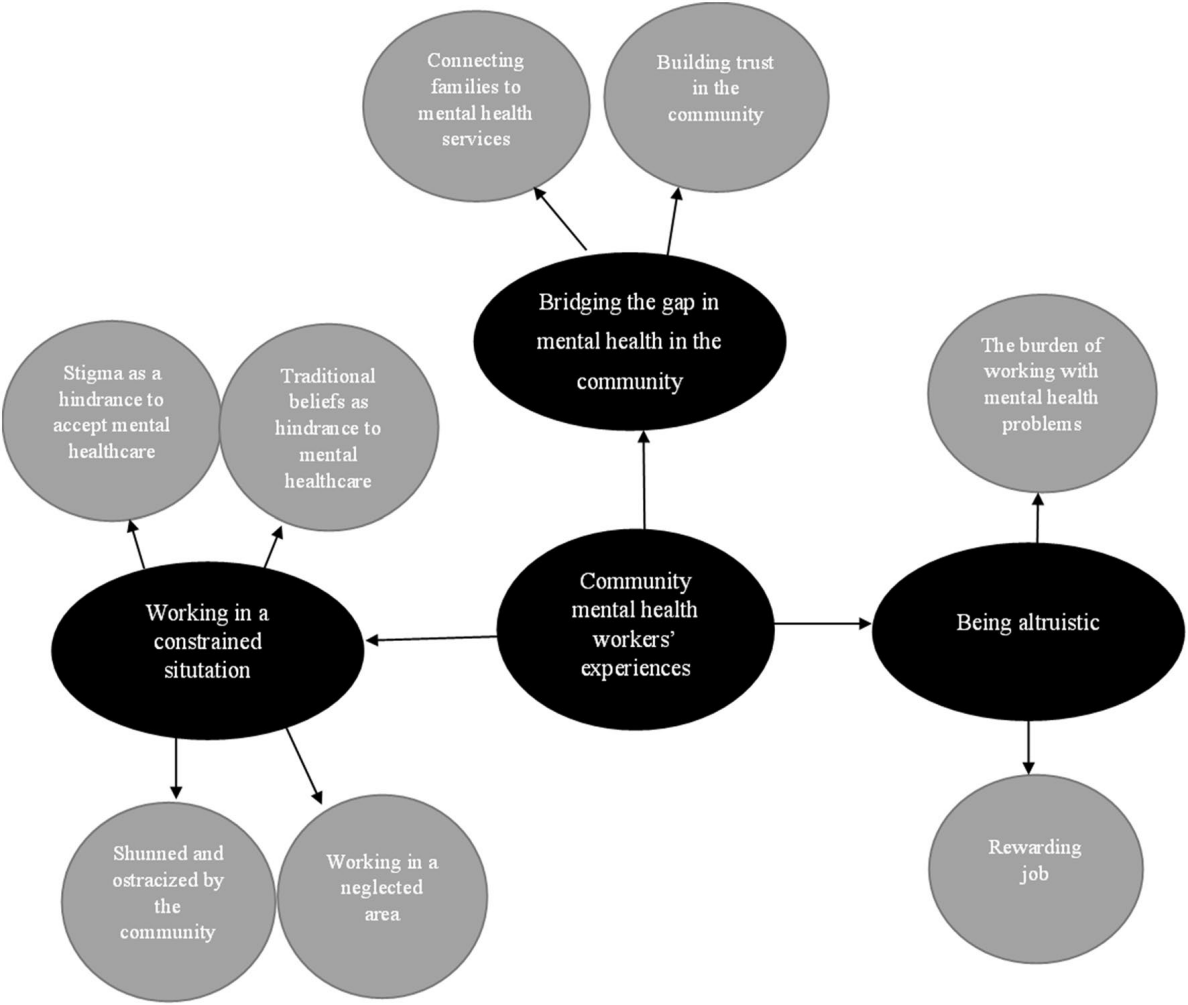
Community mental health experiences on delivering mental health services in the community

The following three categories were identified in the analysis: (1) bridging the mental health gap in the community; (2) working in a constrained situation; and (3) being altruistic. The findings indicated that the FCHWs, doctors, and nurses performed a broad spectrum of roles offering support and showing commitment to their patients. The participants’ statements revealed that they faced some obstacles while providing care to the community, and they further described their working situation as relatively “constrained”. However, the findings also revealed workers’ emotional responses to their roles and that the health services were delivered with benevolence and were appreciated by the patients. The three identified categories and their associated subcategories are presented in Fig. 1. They are also discussed in depth below and illustrated with representative statements by the participants.

### Category 1: Bridging the Gap in Mental Health in the Community

This category reflected how community mental healthcare workers viewed their work as well as the demand and guiding values they experienced.

#### Connecting Families to Mental Health Services

All participants highlighted the stigma and discrimination surrounding mental illness in the community, which resulted in caregivers hiding mentally ill persons in isolation. Initially, it was difficult for the FCHWs to gain access to homes to support families with mentally ill persons. To earn trust in the community, the FCHWs started to sensitize the community about mental health and the importance of seeking care. Although care for newly diagnosed patients was paramount, caregivers and mentally ill persons also required support and information about the care that mentally ill people needed from family and friends. Because most of the recently detected cases of mental illness were either referred to the hospital or treated at home by a psychiatric specialist, the FCHW continued to visit the household and offer support to the families. When medication was prescribed to a patient by the project’s psychiatric doctor, the FCHWs followed up on the case to ensure the patient received the prescribed medication, also providing support and education to the caregivers. The FCHWs not only gave information and motivated the family and patient to seek care themselves but also acted as a bridge between the patient and caregivers and the project doctors, project nurses, and health professionals in the hospital. A female health workers stated:

If you are helping a patient, you are going to their home, taking them to the hospital, and staying with them until the doctor finishes checking the patient. We are not only giving them information, but every morning, we also try to help them and connect them to the health centers and be there for them. (FGD1, R7)

If the family refuses to take the patient to the hospital, we call the doctor, so the patient can get treatment them in their home. (FGD2, R5)

#### Building Trust in the Community

Building trust was important for participants, whether they were FCHWs, doctors, or nurses due to the stigma related to mental health problems. The participants highlighted that young people—particularly girls—were in a vulnerable situation. The most difficult experience that the FCHWs discussed was when families hid their family members, most often girls. The FCHWs described how families chained their daughters in their rooms both because they were ashamed of what the neighbors and community might say and to protect their daughters from abuse of harm. As one female community health worker stated:

Several times, I visited a mother who was hiding her daughter, who was tied with a chain. When the mother came to know that we work for people with mental illnesses and that many of them take medicines and recover, she contacted us later and received our support. Her daughter’s illness was diagnosed by the doctor as bipolar disorder. (FGD1, R4)

According to the participants, some family members and patients were in denial regarding the mental health problems, but knowledge of other patients’ successful outcomes changed their minds and led them to accept seeking the community mental health services that the FCHWs offered. Aside from their devotion to providing mental healthcare services, the FCHWs empathized with the need for financial support of the more vulnerable families who could not afford the medical bills. As the community mental health program did not have sufficient funding to pay for all the medications the patients needed, the FCHWs contributed to paying some medication fees. The participants emphasized that it was part of their role to be compassionate and supportive and not only to make the patients and caregivers aware of the illness, seek care, or accept treatment. Therefore, the FCHWs and nurses came to the support of families when they had, for instance, other socioeconomic problems.

### Category 2: Working in a Constrained Situation

In this category, the participants discussed some of the challenges they experienced in providing care to patients with mental illness and their families.

#### Stigma as a Hindrance to Accept Mental Healthcare

The participants reported that most community members believed that mental illness was not curable, which made their work harder. As one of the participants said, “Some say that medicine gives some relief from the disease, but it does not remove the disease” (FGD3, R5).

Community members would keep the patient tied at home with no proper hygiene and nutrition to control the person because most viewed a person with mental illness as potentially aggressive at any time without any warning. Binding people with mental health problems, such as schizophrenia or/and psychosis, caused them to develop physical complications and lifelong deformity. A lack of knowledge and understanding of mental health conditions resulted in some family members denying the condition’s existence. Despite the FCHWs’ efforts at sensitization, the participants felt that some communities still upheld the belief that mental illness does not exist. The most vulnerable people affected by these beliefs were children with severe mental disorders whose parents denied that their children had mental illnesses and prevented them from taking the prescribed medicine. According to the participants, the parents who rejected the treatment were mostly afraid of what other people might say about their child. As one of the doctor and nurse noted:

There are many children with psychosis problems, but their parents reject the existence of the disease. (FG3, R3)

This case we had … the patient’s uncle took him but came to me again. He said that the boy does not have the disease that you are telling us about, why do you label him as a mentally ill person? (FG3, R2)

#### Traditional Belief as a Hindrance to Mental Healthcare

All FGD participants highlighted that modern medication was not something that community members sought when they or their relatives were suffering from mental illness. According to the participants’ experiences and perceptions, most community members sought traditional and spiritual healers to treat mental illness in the community, which was a hindrance to providing community mental health services. The popularity of traditional healers was due to cultural understanding within the community and the people’s belief that most mental health illnesses were associated with an evil spirit. The participants described how the community trusted traditional healers to cure mental illness, stating that some family members would stop the patient from taking the prescribed medication and prefer them to visit traditional healers for treatment.

Some people take the patient to the Sheikhs. Particularly our elderly people believe in taking the person to the traditional healer’s center. It is argued that the patient’s father was a member of a specific group, so when their father died, it is believed that the father’s spirit moved into his child. (FGD1, R2)

#### Shunned and Ostracized by the Community

The FCHWs felt that some community members shunned them and viewed them as uneducated and inexperienced. The stigma and discrimination surrounding mental illness and the fact that the FCHWs did not have a medical background prompted some community members to forbid the FCHWs from visiting their houses. The participants described this as one of their biggest challenges because even though they had training and were supervised daily by the nurses and doctors, they were not well accepted by the community, particularly in the project’s early stages. This lack of acceptance jeopardized the health and security of the people with mental illnesses whom the FCHWs were expected to refer to the hospital. The FCHWs also reported the challenge of wealthy or middle-class families refusing home visits or their advice. Sometimes, these families claimed that a doctor was already seeing their mentally ill relative. One strategy that the FCHWs applied in certain situations was to bring doctors or nurses with them when carrying out home visits. One female worker noted:

Those who have a better life [middle-class families] do not allow you to knock on their doors or visit them, and maybe they have two or three persons with mental illness at home. (FGD2, R2)

All participants described how they strived to reach the patient and family members to assist them in recovering from their mental illness. However, despite these efforts, some community members undervalued their work, denounced them as liars, and even poured water or spat on them. The FCHWs were accused of both doing the work to earn money at the cost of the relative with the mental illness and giving medications that were making the patient much worse. Some FCHWs also reported facing threats from the patients and sometimes the family. As one of the FCHW said, “she [the mother of the patient] abused us and said that we were making her son mad in reference to the medications being given to the patient. So, there are such obstacles” (FGD2, R3).

#### Working in a Neglected Area

While the participants explained the challenges of performing their duties due to the uncertainties they faced from the community, the greatest challenge they encountered was the lack of resources. For instance, FCHWs walked long distances with no means of transportation to the community to which they provided services. They faced similar difficulties in other related responsibilities, such as sensitizing the community to mental health, referring and linking patients to healthcare personnel, and carrying out house visits and follow-up visits to ensure that the patient was taking their medication. With these numerous responsibilities, the FCHWs felt that the wages attached to the job were minimal. However, they emphasized that the project aimed to rebuild their community after the previous war and conflict, and they wanted to contribute to this rebuilding. The participants stated that the government and other stakeholders neglected the mental health. They felt that the area was underprioritized, with little or no resources spent on mental health prevention or treatment.

The biggest problem is that mental health is not a priority area in Somaliland. (FGD3, R1)

### Category 3: Being Altruistic

In this category, the participants described their empathic view based on humanistic and religious principles. They described themselves as being overprotective of and affectionate and warm towards people with mental health problems. Being caring, spiritual, and compassionate towards people with mental illnesses and their families were some of the diverse attitudes that characterized the moral viewpoints of the participants. They also highlighted that even though their work contained a significant burden, it was also rewarding.

#### The Burden of Working with Mental Health Problems

The participants stated that they had many responsibilities, which required substantial effort and time. In most cases, they even went an extra mile to give their patients the best care they could. They emphasized that as Somaliland does not have many nurses and doctors with psychiatric backgrounds, the FCHW training had many benefits, not only serving as a link between healthcare providers and the community but also taking the burden from the doctors and nurses.

The female community health workers do more work compared to others. She [female health worker] finds the patient, connects to the doctor. She is required to bring patients and follow up on their situation. They work in a very large area. The number of female health workers in Borama is 20, yet they have a very low salary. The job requirements put pressure on the women to work hard. (FGD3, R3)

The participants reported that working with mental health problems might cause them stress because the issues are complex and they must deal with policy makers, managers, and the community, as well as traditional healers. The participants requested training for and support in their daily work.

#### Rewarding Job

Despite the challenges they faced in the community and the hardships they encountered in executing their duties, the participants felt motivated to carry on after seeing patients recover from their mental illnesses through the services they rendered. Due to their Islamic beliefs, they also thought that the job was rewarding. They believed that helping a person with a mental illness is a humane act and that their work would be rewarded both in this life and the next.

My job is good. The best reward in both this life and after is to help those who are mentally ill. It is humane to work for poor people who have no other person helping them. (FGD3, R1)

According to the FCHWs, the work gave them not only a sense of reward but also the motivation and inspiration to pursue their dreams of continuing their education. Seven of the 22 FCHWs continued on to higher education, and other FCHWs were planning to do so.

## Discussion

This study explored the experiences of FCHWs, doctors, and nurses in providing mental health services to the community. The study was part of a larger project implementing community mental health services in the Awdal region in Somaliland. The project was the first of its kind in the Somali region to tackle mental health problems by engaging FCHWs, in collaboration with doctors and nurses. Our findings showed that community health workers understood their role was to bridge the mental health gap in the community. They maintained that community mental healthcare was strangled due to the ostracization of and traditional beliefs about mental problems, which served as obstacles to providing mental health services. A lack of support from the government and other stakeholders was another challenging aspect of the FCHWs’ work. However, they felt benevolent toward their patients and worked hard despite the challenges, making a patient-centered approach their main objective.

The findings of the study suggested that FCHWs had more challenges regarding their work compared to doctors and nurses working on the same project. They struggled to be recognized as community health workers due to their lack of healthcare education. Similar challenges have been reported in previous studies (George, 2008; Sarin & Lunsford, 2017). Despite the challenges that the community mental health workers in this study reported, they felt motivated to care for people with mental health problems. They highlighted that their faith in God kept them strong in continuing to render their services to the community because they felt that the work was rewarding. This finding is in line with several studies that community mental health workers perceived the significance of their work as extending beyond the prescribed job description to duties outside their scope (Agyapong et al., 2015; Ozcan et al., 2021). Many of them highlighted their faith as a motivator and driver to serve vulnerable people (Ozcan et al., 2021). The issue of community health workers being altruistic can be seen in the light of stress-buster and preventing them from burn-out (Chow et al., 2021).

The FCHWs benefited from participating in the overall project and became more confident and motivated to continue with higher education. Increased self-efficacy among FCHWs while implementing community health services was also reported in a previous study (Sarin & Lunsford, 2017). Although, the FCHWs received weekly supervision, they emphasized they needed more training in order to feel confident for the work they are doing. One potential area where the FCHWs could be trained is in the development and implementation of tools to screen people they suspect having mental health problems. For example, FCHWs in this study asked the households if there was someone with mental illness or observed anyone in a chain. Therefore, training them on mental health screening tools is needed. More importantly, culturally developing or validating existing screening tools for mental health illness would guide FCHWs to refer the patient to healthcare (Allden et al., 2009; Kaiser et al., 2019).

Although the participants in our study experienced their role as bridging the gap, they felt that the community frequently turned to traditional healers. One reason for the community’s preference for traditional healers was that the healers delivered care to places near where people lived and worked, improving the accessibility of the service they offered. In addition, traditional healers explain the reason for mental health problems when people seek care from them, which might increase their credibility in the community (Akol et al., 2018; Baumgartner et al., 2015). Traditional healers can become a therapeutic ally in community mental health system (Akol et al., 2018). However, studies reported the mutual lack of trust between the healers and the modern health practitioner (Akol et al., 2018; Burns & Tomita, 2015). Therefore, it is crucial to find innovative way of how traditional healers and modern health practitioner could collaborate without hampering the safety of people with mental health problems (Akol et al., 2018; Burns & Tomita, 2015; Pouchly, 2012).

Another challenge for the community health workers in our study to bridge the gap for the community to seek mental healthcare was the stigma and attitudes surrounding mental illness (Asher et al., 2017; Marrow & Luhrmann, 2012). A reduction in the public stigma attached to mental illness is needed to promote the patient’s integration into society (Kakuma et al., 2010). Several studies have highlighted the fact that stigma is a barrier to help seeking, creates poor access to mental health services, and contributes greatly to mental health treatment gaps. These gaps exist principally in low- and middle-income countries, making it crucial to identify effective and efficient strategies to reduce stigma in those settings (Arthur et al., 2020; Kaur et al., 2020). Collaboration between traditional healers and modern medical practitioners on awareness about mental health could possibly reduce the levels of stigma and discrimination (Pouchly, 2012).

Similar to our findings, a study conducted in Ethiopia showed that families hid and chained their family member with mental illness due to shame but also to protect the person (Asher et al., 2017). This chaining leads to human rights violations for people with mental illness (Asher et al., 2017). According to the FCHWs in our study, community perceptions of and the lack of knowledge about the causes and treatment of mental health problems posed a challenge for the FCHWs to perform their work as expected (Labinjo et al., 2020). Increasing community awareness as well as engaging people who have recovered from mental illness is important to implementing health education and prevention of mental health (Eaton et al., 2017). Using community health workers to reduce stigma around mental illness has been shown to have a positive impact (Abdi et al., 2021; Eaton et al., 2017).

Our findings indicated that the participants worked in a strenuous situation due to there being little or no resources dedicated to mental health to support mental health workers in managing mentally ill patients both at the community and hospital levels. This is the case not only in Somaliland but also in other African countries, where less than 1% of the health budget is spent on mental health services, and most of the money goes to urban based mental health institutions (Cole & Tembo, 2011). Furthermore, people who are already reluctant to seek help for a mental health problem because of stigma might be forced to delay treatment until their needs are acute due to the high cost of out-of-pocket payments, making the necessary care even more expensive and potentially catastrophic. In our study, the participants reported that they paid some of the patient’s medical treatment out of their own pocket, which is not a sustainable solution.

Systematic decentralization of mental healthcare to the community level has long been recommended by WHO. Whether this model is feasible in the context of Somaliland should be further explored. Recently, the Ministry of Health and Development (MOHD) in Somaliland developed a mental health strategic plan to reduce the gap between mental health problems and the service provided (Setchell, 2022). Somaliland’s MOHD is trying to find solutions to address human resource shortages to deliver mental health interventions.

## Strengths and Limitations

The study has certain strengths and limitations that should be outlined. One strength is that data were collected data from all staff (FCHWs, doctors, and nurses) who implemented the community mental health services in the Awdal region, Somaliland. To ensure the credibility and confirmability of the findings, the first and last authors performed the analysis together. The FGDs were conducted in the participants’ own native language, which has the advantage of making it possible to obtain firsthand information from the participants without missing details, contributing to rich data. However, it can also lead to the moderator (last author) missing or failing to ask follow-up questions because of a possible insider perspective. However, the moderator was experienced in leading FGDs, and the use of the interview guide contributed to the credibility of the study and ensured that the discussions aligned with the aim of the study. Another strength is that the moderator and first author were not involved in the implementation of community mental health services in Somaliland. Instead, they conducted the study as external evaluators. One potential limitation is that the interview guide was not pilot tested prior to conducting the FGDs. This was due to the studied project being the only community-based mental health service in Somaliland at the time of the study. Nonetheless, the interview guide was discussed in the advisor committee for the project.

The study findings must be considered in light of all the participants being from one region and being involved in the implementation of the project. However, findings can be transferred to similar cultural traditional belief system related to mental health issues, since they may also face ‘health system challenges in the backdrop of a post-conflict status.

## Conclusion

The study highlighted several challenges that the community health workers faced. Importantly, it also showed the importance of having FCHWs to bridge the gap in the mental health system and that an enabling work environment is crucial to maximizing the productivity of community healthcare workers. Policymakers and stakeholders need to allocate adequate funding and organize periodic training to FCHWs, as well as build on their altruistic motives to take quality mental health care to the community, which may motivate other healthcare providers to integrate mental health services in their area of operation. Participants in our study highlighted that the community frequently turned to traditional healers. For this reason, it is crucial to find innovative way of how traditional healers and modern health practitioner could collaborate without hampering the safety of the patient. This current project has only been driven by the community and a nongovernmental organization; therefore, it needs to be incorporated into the wider healthcare system.

## Data Availability

The data are not open access. Data sharing is possible upon request after assessment from the research group and ethical approval.

## Abbreviations

FCHW: Female community health worker
FGD: Focus group discussion
WHO: World Health Organization

## References

[CR1] Abdi YA, Said NI, Hared YA, Ayeh I, Walhad SA (2021). Mental health care delivery in poor settings through trained female community health workers: A five-year intervention program in Somaliland. Somali Health Action Journal.

[CR2] Abdi Abdillahi F, Ismail EA, Singh SP (2020). Mental health in Somaliland: A critical situation. BJPsych International.

[CR3] Agyapong VI, Osei A, Farren CK, McAuliffe E (2015). Task shifting–Ghana’s community mental health workers’ experiences and perceptions of their roles and scope of practice. Global Health Action.

[CR4] Akol A, Moland KM, Babirye JN, Engebretsen IMS (2018). We are like co-wives”: traditional healers’ views on collaborating with the formal child and adolescent mental health system in Uganda. BMC Health Services Research.

[CR5] Allden K, Jones L, Weissbecker I, Wessells M, Bolton P, Betancourt TS, Sumathipala A (2009). Mental health and psychosocial support in crisis and conflict: report of the mental health working Group. Prehosp Disaster Medicine.

[CR6] Arthur YA, Boardman GH, Morgan AJ, McCann TV (2020). Effectiveness of a problem-solving, story-bridge mental health literacy programme in improving Ghanaian community leaders’ attitudes towards people with mental illness: A cluster randomised controlled trial. Issues in Mental Health Nursing.

[CR7] Asher L, Fekadu A, Teferra S, De Silva M, Pathare S, Hanlon C (2017). "I cry every day and night, I have my son tied in chains”: physical restraint of people with schizophrenia in community settings in Ethiopia. Globalization and Health.

[CR8] Baumgartner JN, Kaaya S, Siril H (2015). Mental health screenings for couples at churches in Nigeria: A strategy for enhancing community-based maternal mental health services in low-resource settings. Social Psychiatry and Psychiatric Epidemiology.

[CR9] Burns JK, Tomita A (2015). Traditional and religious healers in the pathway to care for people with mental disorders in Africa: A systematic review and meta-analysis. Social Psychiatry and Psychiatric Epidemiology.

[CR10] Campion J, Javed A, Lund C, Sartorius N, Saxena S, Marmot M, Udomratn P (2022). Public mental health: Required actions to address implementation failure in the context of COVID-19. The Lancet Psychiatry.

[CR11] Chaulagain A, Pacione L, Abdulmalik J, Hughes P, Oksana K, Chumak S, Gasparyan K (2020). Who mental health gap action programme intervention guide (mhGAP-IG): The first pre-service training study. International Journal of Mental Health Systems.

[CR12] Chow SK, Francis B, Ng YH, Naim N, Beh HC, Ariffin MAA, Sulaiman AH (2021). Religious coping, depression and anxiety among healthcare workers during the COVID-19 pandemic: A malaysian perspective. Healthcare (Basel).

[CR13] Cole SM, Tembo G (2011). The effect of food insecurity on mental health: Panel evidence from rural Zambia. Social Science & Medicine.

[CR14] Dattani, S., Ritchie, H., & Roser, M. (2021). Mental Health. *Our World in Data*. Retrieved from https://doi.org/https://ourworldindata.org/mental-health

[CR15] Eaton J, Nwefoh E, Okafor G, Onyeonoro U, Nwaubani K, Henderson C (2017). Interventions to increase use of services; Mental health awareness in Nigeria. International Journal of Mental Health Systems.

[CR16] Elo S, Kääriäinen M, Kanste O, Pölkki T, Utriainen K, Kyngäs H (2014). Qualitative content analysis: A focus on trustworthiness. Sage Open.

[CR17] Frankish H, Boyce N, Horton R (2018). Mental health for all: A global goal. The lancet.

[CR18] George A (2008). Nurses, community health workers, and home carers: Gendered human resources compensating for skewed health systems. Global Public Health.

[CR19] Handuleh JI, Gurgurte AM, Elmi A, Aye IM, Abubakr F, Kayd MA, Abdi YA (2014). Mental health services provision in Somaliland. The Lancet Psychiatry.

[CR20] Ibrahim M, Rizwan H, Afzal M, Malik MR (2022). Mental health crisis in Somalia: A review and a way forward. International Journal of Mental Health Systems.

[CR21] Iheanacho T, Kapadia D, Ezeanolue CO, Osuji AA, Ogidi AG, Ike A, Ezeanolue EE (2016). Attitudes and beliefs about mental illness among church-based lay health workers: Experience from a prevention of mother-to-child HIV transmission trial in Nigeria. International Journal of Culture and Mental Health.

[CR22] Kaiser BN, Ticao C, Anoje C, Minto J, Boglosa J, Kohrt BA (2019). Adapting culturally appropriate mental health screening tools for use among conflict-affected and other vulnerable adolescents in Nigeria. Global Mental Health (Cambridge England).

[CR23] Kakuma R, Kleintjes S, Lund C, Drew N, Green A, Flisher A (2010). Mental health stigma: What is being done to raise awareness and reduce stigma in South Africa?. African Journal of Psychiatry.

[CR24] Kaur A, Kallakuri S, Kohrt BA, Heim E, Gronholm PC, Thornicroft G, Maulik PK (2020). Systematic review of interventions to reduce mental health stigma in India. Asian Journal of Psychiatry.

[CR25] Labinjo T, Serrant L, Ashmore R, Turner J (2020). Perceptions, attitudes and cultural understandings of mental health in Nigeria: a scoping review of published literature. Mental Health Religion & Culture.

[CR26] Leather A, Ismail EA, Ali R, Abdi YA, Abby MH, Gulaid SA, Lowe-Lauri M (2006). Working together to rebuild health care in post-conflict Somaliland. The lancet.

[CR27] Lindvall K, Kinsman J, Abraha A, Dalmar A, Abdullahi MF, Godefay H, Musumba J (2020). Health status and health care needs of drought-related migrants in the Horn of Africa—a qualitative investigation. International Journal of Environmental Research and Public Health.

[CR28] Marrow J, Luhrmann TM (2012). The zone of social abandonment in cultural geography: On the street in the United States, inside the family in India. Culture Medicine and Psychiatry.

[CR29] Ozcan O, Hoelterhoff M, Wylie E (2021). Faith and spirituality as psychological coping mechanism among female aid workers: A qualitative study. Journal of International Humanitarian Action.

[CR30] Patel V, Garrison P, de Jesus Mari J, Minas H, Prince M, Saxena S (2008). The Lancet’s series on global mental health: 1 year on. The Lancet.

[CR31] Patel V, Saxena S, Lund C, Thornicroft G, Baingana F, Bolton P, Eaton J (2018). The Lancet commission on global mental health and sustainable development. The Lancet.

[CR32] Pouchly CA (2012). A narrative review: Arguments for a collaborative approach in mental health between traditional healers and clinicians regarding spiritual beliefs. Mental Health Religion & Culture.

[CR33] Rivelli F (2010). A situation analysis of mental health in Somalia.

[CR34] Rossi A, Cetrano G, Pertile R, Rabbi L, Donisi V, Grigoletti L, Amaddeo F (2012). Burnout, compassion fatigue, and compassion satisfaction among staff in community-based mental health services. Psychiatry Research.

[CR35] Rössler W (2012). Stress, burnout, and job dissatisfaction in mental health workers. European Archives of Psychiatry and Clinical Neuroscience.

[CR36] Sarin E, Lunsford SS (2017). How female community health workers navigate work challenges and why there are still gaps in their performance: A look at female community health workers in maternal and child health in two indian districts through a reciprocal determinism framework. Human Resources for Health.

[CR37] Setchell, C. (2022). *Reducing the gap in mental health services in Somaliland*. Retrieved from https://www.kcl.ac.uk/reducing-the-gap-in-mental-health-services-in-somaliland

[CR38] Thornicroft G, Deb T, Henderson C (2016). Community mental health care worldwide: Current status and further developments. World Psychiatry.

[CR39] United Nations Population Fund, S. C. o (2014). *Population estimation survey—for the 18 pre-war regions of Somalia*. Retrieved from Nairobi, Kenya. https://www.unfpa.org

[CR40] WHO (2015). Update of the Mental Health Gap Action Programme (mhGAP) guidelines for mental, neurological and substance use disorders, 2015.

